# Quantifying yeast lipidomics by high-performance thin-layer chromatography (HPTLC) and comparison to mass spectrometry-based shotgun lipidomics

**DOI:** 10.15698/mic2024.02.815

**Published:** 2024-02-21

**Authors:** Thorsten Meyer, Oskar Knittelfelder, Martin Smolnig, Patrick Rockenfeller

**Affiliations:** 1 Chair of Biochemistry and Molecular Medicine, Center for Biomedical Education and Research (ZBAF), University of Witten/Herdecke (UW/H), Stockumer Str. 10, 58453 Witten, Germany.; 2 Max Planck Institute of Molecular Cell Biology and Genetics, 01307 Dresden, Germany.

**Keywords:** lipidomics, lipid metabolism, lipid analysis, HPTLC, thin-layer chromatography

## Abstract

Lipidomic analysis in diverse biological settings has become a frequent tool to increase our understanding of the processes of life. Cellular lipids play important roles not only as being the main components of cellular membranes, but also in the regulation of cell homeostasis as lipid signaling molecules. Yeast has been harnessed for biomedical research based on its good conservation of genetics and fundamental cell organisation principles and molecular pathways. Further application in so-called humanised yeast models have been developed which take advantage of yeast as providing the basics of a living cell with full control over heterologous expression. Here we present evidence that high-performance thin-layer chromatography (HPTLC) represents an effective alternative to replace cost intensive mass spectrometry-based lipidomic analyses. We provide statistical comparison of identical samples by both methods, which support the use of HPTLC for quantitative analysis of the main yeast lipid classes.

## INTRODUCTION

The analysis of cellular lipidomics as a powerful tool to characterise cells or cellular states has gained a lot of attention throughout the last two decades [1]. Mass spectrometry-(MS) assisted lipidomic analyses have been developed as a routine to analyse lipidomes of basically any cell type, tissue or cell-organelle from any species [2–6]. Importantly, lipidomic changes can be associated with ageing [7–9] or manifest in a diversity of diseases. These include neurodegenerative diseases such as Alzheimer's disease and dementia [10] or cardiovascular diseases [11]. Lipidomics are further key to investigate lipotoxicity mechanisms which are fundamental to lipid-related diseases [12]. Hence lipidomics also offer potential use as diagnostic markers of disease and ageing.

The budding yeast *Saccharomyces cerevisiae* is used as a model organism in scientific research because of several reasons: It is easy to grow at large quantities, cultures are clonogenic, genetic modifications are easy to perform and an ever-growing number of yeast libraries [13] is available. *S. cerevisiae* has been harnessed to study cellular processes such as autophagy [14–19], ageing [20–25], cell death [26–30], lipid metabolism [31, 32], lipid droplet (LD) dynamics [33–36], lipotoxicity [26, 37–39], actin dynamics [40–44], vesicle traffic [45–48] and many others. A connecting feature of the before-mentioned cellular processes is the importance of cellular lipid analysis. Changes of cellular lipid homeostasis can significantly contribute to the regulation of cell health and death, which makes lipidomic analysis an important tool for the research community.

MS-assisted lipidomics are very sensitive and deliver precise pictures of lipidomes. One draw-back of MS-assisted lipidomics, however, is the need of expensive MS equipment including established lipidomic protocols or, alternatively if outsourced, high costs for lipidomic services offered by commercial companies. High-performance thin-layer chromatography (HPTLC) represents a low-cost alternative for lipidomic measurements. The method is not as sensitive as MS-assisted lipidomics but is suitable to detect the major neutral lipid and phospholipid classes especially if availability of sample material is not a limiting factor. Simple thin-layer chromatography (TLC) has been used extensively in the past for lipid quantification [49–54]. Importantly, HPTLC has emerged as a more powerful advancement of the technique, which is mainly achieved through the use of improved HPTLC plates. HPTLC plates still mostly contain silica gel coatings such as conventional TLC plates, but the plates are generally smaller, contain pre-coatings with smaller particle size and narrower particle size distribution [55]. The plates further contain thinner layers and the surface is smoother [55]. These features altogether reduce sample quantity requirements and allow for more economical and faster separation with reduced diffusion rates [55–57]. This results in higher sensitivity, more efficient separation and better sample resolution, and also positively affects reproducibility [55–57].

Here we describe a method to analyse basic lipidomics in yeast using HPTLC and offer comparison to measurement of the same samples with MS-based shotgun lipidomics. We further validate the lipidomic quantification method using two established yeast knock out models (1. *dga1*Δ *lro1*Δ *are1*Δ *are2*Δ (quadruple knock out; QKO), and 2. *pah1*Δ) both bearing characteristic impacts on lipid metabolism.

## RESULTS AND DISCUSSION

We aimed at establishing a standard protocol for HPTLC-based quantitative lipidomics in yeast. Since no one-dimensional separation system with a single solvent mixture is capable to fully separate neutral- and phospholipids in one run, we decided to separate the neutral lipids with n-hexane, n-heptane, diethylether, acetic acid (63/18.5/18.5/1 v/v/v/v) as mobile phase [58], whereas phospholipid separation was carried out using chlorofom (CHCl_3_), methanol (MeOH) and distilled water (dH_2_O) (32.5/12.5/2; v/v/v) as mobile phase [49, 53, 54]. Unmodified yeast lipids are invisible on HPTLC plates. We thus compared a number of common derivatisation procedures to visualise the lipid classes [49, 57]. The most commonly used procedures for non-specific lipid derivatisation include carbonisation (also called charring) by heating after spraying with 10-50% H_2_SO_4_ or MnCl_2_. These derivatisation steps irreversibly alter the lipid moieties which can be problematic if further analysis of the lipids is desired. Reversible staining with iodine vapor or usage of the fluorescent dye primuline are alternative non-destructive methods for visualisation [56, 57]. We decided to use the fluorophore primuline as a revealing agent which in fact is not a derivatising agent as it only involves non-covalent interaction of the fluorophore with the lipid [57]. Primuline unspecifically interacts with the lipid hydrocarbon chains by dipole-induced dipole interaction. This interaction with long hydrocarbon chains leads to an increase in Primuline's-fluorescence intensity [59]. Primuline-based detection offers good sensitivity while at the same time not being limited to unsaturated or saturated fatty acid moieties. However, the fluorescence intensity signal may vary upon the hydrocarbon chain length.

### 1. Establishing suitable lipid standards for neutral and phospholipid analysis

We first established standard mixtures of pure single component lipids for neutral and phospholipid separation. We tried to select lipid components, which structurally relate the most to yeast lipids with regard to carbon chain length and saturation/unsaturation-level. Thus, we chose substances containing palmitate (16:0), palmitoleate (16:1), stearate (18:0) and oleate (18:1), which are the predominant acyl-moieties in *S. cerevisiae* [31, 60, 61]. We reasoned that our choice of lipids in the standard emulates the main components of the yeast lipidome to allow for an accurate quantification of the main lipid classes.

**Figure 1:**
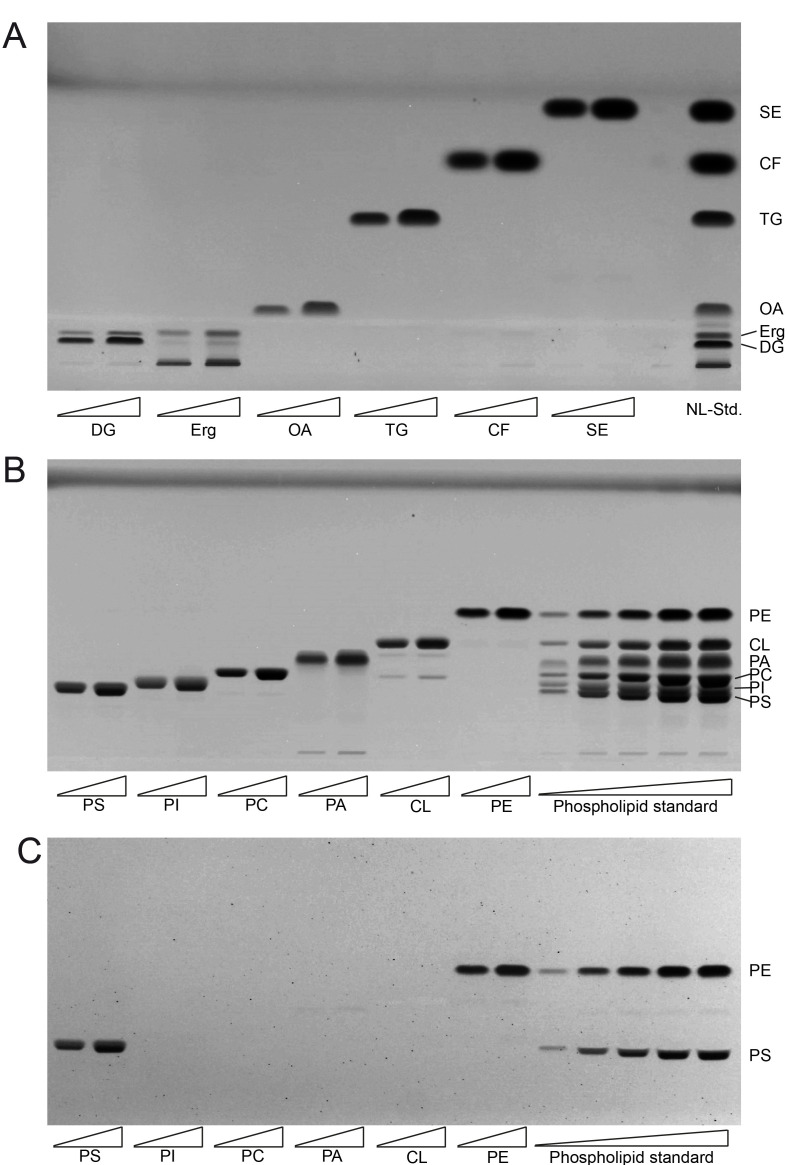
FIGURE 1: Establishing lipid standards for HPTLC. **(A)** Developed HPTLC plate showing single components of neutral lipid standard (NL-Std.) with an absolute input of 5 µg (left) and 10 µg (right). **(B)** Developed HPTLC plate showing single components of phospholipid standard (PL-Std.) with an absolute input of 5 µg (left) and 10 µg (right). In the final five lanes the whole PL-Std.-mixture was applied at increasing concentrations from 500 ng to 12.5 µg absolute mass. **(C)** The developed HPTLC-plate from panel B was derivatised using ninhydrin to visualise PS and PE only. FA, fatty acid; TG, triglyceride; CF, cholesterylformate; SE, sterol ester; Erg, ergosterol; DG, diglyceride, PI, phosphatidylinositol; PC, phosphatidylcholine; PE, phosphatidylethanolamine; PS, phosphatidylserine; CL, cardiolipin; PA, phosphatidic acid.

The single component TLC run of the neutral lipid standard shows single bands for almost all of the components which include oleic acid (OA) as representative for fatty acids (FA), triolein as representative for triglycerides (TG), cholesterylformate (CF), which was used as internal standard, and cholesteryl-oleate as representative sterol ester (SE; **Fig. 1A**). However, ergosterol (Erg) unexpectedly delivered two bands, one at the expected Rf distance and an additional one at the origin of application, which was considered as impurity and thus ignored. As diglyceride (DG) standard we used 1-palmitoyl-2-oleoyl-sn-glycerol, which also delivered two bands. The main band at the bottom was considered to correctly relate to 1-palmitoyl-2-oleoyl-sn-glycerol, whereas the upward-shifted second band at lower intensity was considered to be a result of limited acyl-migration yielding 1,3-derivatives. Since this second band is not properly separated from the Erg band, which runs at approximately the same height, the results for Erg are to be used cautiously or better be analysed in a separate run. The use of an alternative Erg product should also be considered to get rid of the unspecific band at the origin of application.

The single component standard of phospholipids included phosphatidylethanolamine (PE), cardiolipin (CL), phosphatidic acid (PA), phosphatidylcholine (PC), phosphatidylinositol (PI), and phosphatidylserine (PS). HPTLC-based separation of the individual standards at two concentrations revealed one major band representative for each phospholipid (**Fig. 1B**). Additional minor bands were only observed at very low densities which was the case for PA (at the origin of application) and for CL (at the approximate Rf-values corresponding to PA and PC). The combined mixture of PL (phosphor lipid)-standards could be separated efficiently at low PL-input levels. At higher application concentrations a full separation of PI and PS could not be achieved. Additional derivatisation with ninhydrine however revealed single bands for PE and PS only (**Fig. 1C**). Derivatisation with ninhydrine was thus considered mandatory to allow for precise PS quantification at high concentrations, excluding any PI in our HPTLC-system.

### 2. All major lipid classes of whole cell yeast extracts can be separated and quantified in two separate runs for neutral and phospholipid analysis using HPTLC

**Figure 2:**
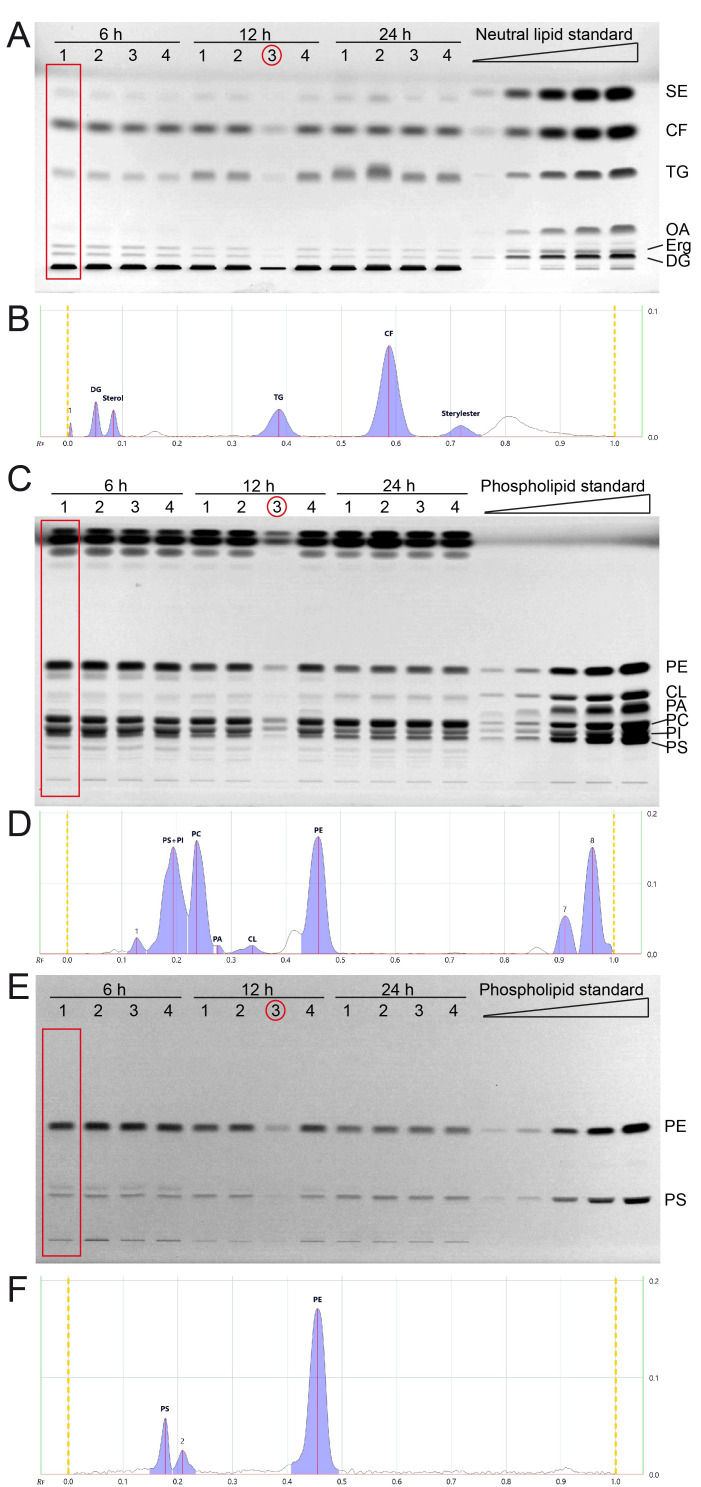
FIGURE 2: All major lipid classes of whole cell yeast extracts can be separated and quantified in two separate runs for neutral and phospholipid analysis using HPTLC. **(A)** Neutral lipids were separated by HPTLC with a mobile phase consisting of n-hexane, n-heptane, diethylether, and acetic acid (63/18.5/18.5/1 v/v). Lipids were derivatised using primuline. **(B)** Example chromatogram with peak integration of lane 1, corresponding to the red rectangle in panel A (Wt 1, 6 h) **(C, E)** HPTLC plate showing phospholipid separation, sequentially derivatised using Primulin **(C)** to visualize all lipids and then applying ninhydrin **(E)** to visualise lipids containing free amino-groups such as PE and PS. As an example of chromatogram peak integration, representative chromatograms of lane 1 from (C) and (E) are shown in **(D)** and **(F)**, respectively. *During the lipid preparation of the third replicate of the 12 h condition (indicated by red circle) material was lost, which resulted in lower overall concentration/yield.

We next cultivated yeast in synthetic complete medium with 2% glucose and additional inositol (SCD+Ino; see note 2) for 6, 12 and 24 hours and extracted the total lipids by Folch extraction according to our standard protocol. Neutral lipids and phospholipids were separated in two distinct HPTLC analyses runs as described in results part 1 and in the methods section. All neutral lipids which include SE, CF (internal standard), TG, OA, Erg, and DG could be separated efficiently using the yeast lipid extracts at any tested timepoint and attributed to references in the neutral lipid standard (**Fig. 2A, B**). OA-levels are at the detection limit with a total sample application volume of 20 µl, but efficient separation is documented in the 6-hour-samples, where OA-levels are highest.

The phospholipids PE, CL, PA, and PC could be separated efficiently using the developing system described in the methods section (**Fig. 2C, D**). PI and PS could only be separated at lower concentrations, which is the case for samples harvested at 12 and 24 hours. The 6 hour-samples have higher PI (and PS) levels which results in inefficient peak separation (**Fig. 2D**). However, additional derivatisation with ninhydrin, which visualises compounds containing free amino groups, was used to additionally measure PS and PE (**Fig. 2E, F**). This ninhydrin-based measurement of PS can be used to separately quantify PS without PI and subsequent calculations deliver the remaining values for PI as well. We further noticed a third band on the ninhydrin-derivatised plates (**Fig. 2E**) which relates to peak 2 in the chromatogram (**Fig. 2F**), which runs slightly further than the main PS band, and is best visible in the 6 h samples. We reason that this band is representative for PS-species or derivatives which occur at lower abundance in yeast.

### 3. & 4. Comparison of HPTLC-based lipidomic quantification of major lipid classes to MS-based shotgun lipidomics

**Figure 3:**
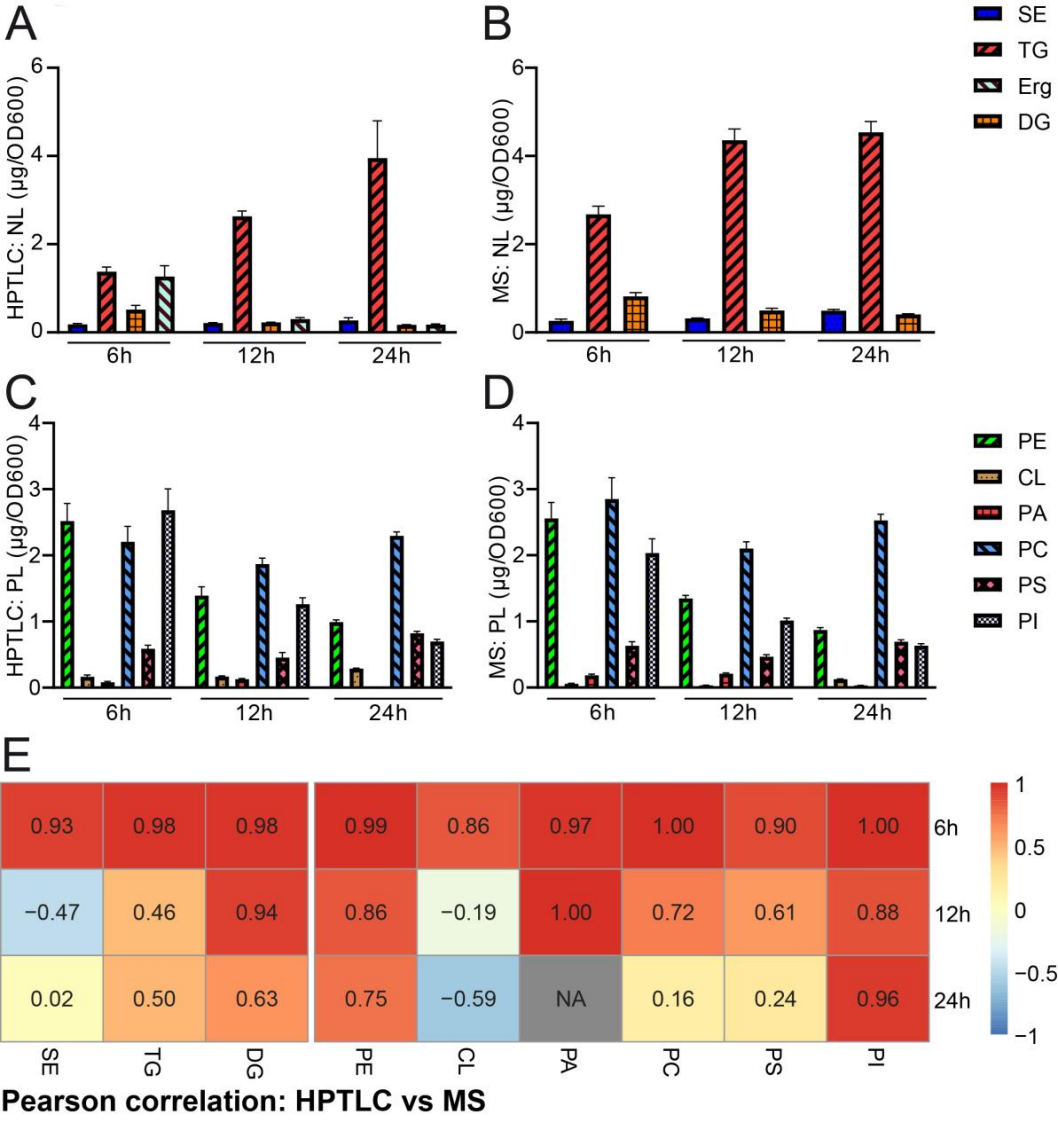
FIGURE 3: Comparative quantification of HPTLC-lipidomics and MS-assisted shotgun lipidomics. **(A, B)** HPTLC-derived neutral lipidomic quantification **(A)** and analysis of the same samples by MS-based shotgun lipidomics **(B)** deliver comparable results regarding absolute amounts of total neutral lipids in yeast. **(C, D)** HPTLC-derived phospholipidomic quantification **(C)** and analysis of the same samples by shotgun lipidomics **(D)** deliver comparable results regarding absolute amounts of the major phospholipid classes in yeast. **(E)** Pearson correlation analysis comparing HPTLC with MS results. Additional relative data quantification in mol % is depicted in Fig. S1 and scatter plots visualising individual pairs for Pearson correlation are shown in Fig. S2.

We next wanted to quantify and compare the results obtained by HPTLC-based lipidomics with MS-based shotgun lipidomic analyses of the exact same samples. The samples were sent to Lipotype, a company which commercially offers lipidomic analyses. The data obtained from lipotype were converted from molar concentrations into absolute mass concentrations according to our conversion table (accessible at Mendelay data) to allow for direct comparison with HPTLC results. The bulk graphical representations of both analyses deliver a similar overall picture for neutral (**Fig. 3A, B**) and phospholipid (**Fig. 3C, D**) quantities, which suggests good comparability of both methods for lipid analysis (see also note 3). Please note that a relative representation of these data is given in the supplemental data (Fig. S1A-D). A Pearson correlation analysis of absolute data pairs attested highest correlation at the 6 h timepoint. The correlation analysis further suggests that comparability is most robust for PA (except for the 24 h timepoint, where PA was beyond detection limit with HPTLC), PI, PE, and DG at all tested time points and PC and PS at 6 h and 12 h time points (**Fig. 3E**, Fig. S2). An analysis of Pearson correlation using the relative datasets as shown in the supplemental data (Fig. S1E) suggests highest correlation for TG and DG at all tested time points, PE at 6 h and 24 h, CL at 12 h, PA at 6 h and 12 h and PS at 12 h.

**Figure 4:**
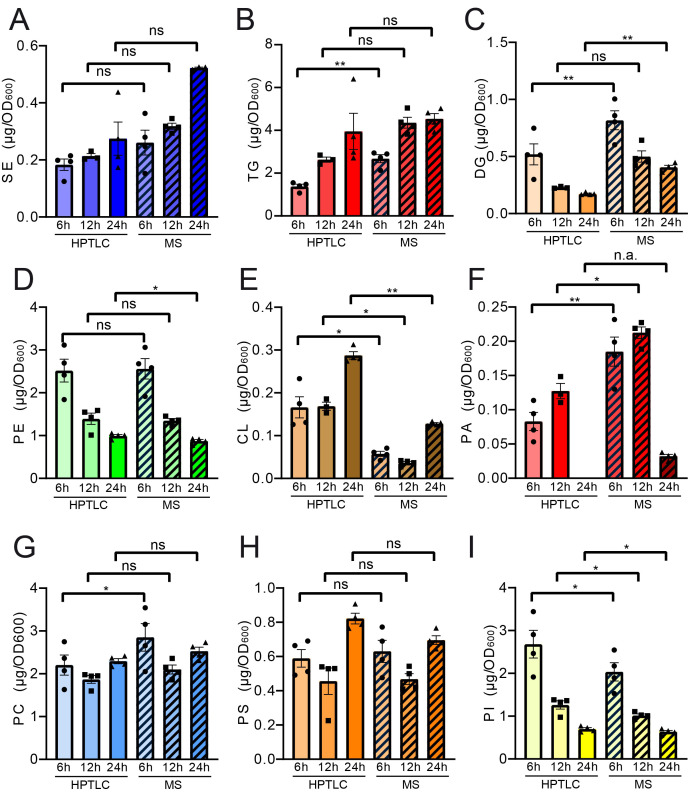
FIGURE 4: Paired statistical analysis of individual lipid classes (absolute) for HPTLC-lipidomics vs. MS-assisted shotgun lipidomics reveals similarities and differences between both methods. Neutral lipids are compared in panels **(A-C)** and phospholipid comparisons are given in panels **(D-I)**. Statistical analysis was performed using paired tests: TG, DG, SE, CL, PA, PC, PS and PI were analysed using mixed effects analysis with Sidaks's multiple comparisons test; PE was analysed using RM ANOVA with Holm-Sidak's multiple comparisons test. Relative representation in mol % is depicted in Figure S3.

Pairwise statistical testing for significant difference between both measurements was assessed for individual lipid classes comparing the absolute values (**Fig. 4 A–I**) and relative values (Fig. S3 A-I). The absolute results for SE (**Fig. 4A**) and PS (**Fig. 4H**) do not differ significantly at any tested timepoints no matter which detection method was used (HPTLC or MS). Likewise, TG (**Fig. 4B**) and PC (**Fig. 4G**) results do not significantly differ at 12 h and 24 h time points. Absolute PE (**Fig. 4D**) comparisons are best at 6 h and 12 h showing no statistical differences between both methods. Statistical differences however were detected for TG at 6 h (**Fig. 4B**), DG at 6 h and 24 h (**Fig. 4C**), PE at 24 h (**Fig. 4D**), CL at all timepoints (**Fig. 4E**), PA at 6 h and 12 h, PC at 6 h and PI at all tested timepoints. We noted that DG quantification delivered higher values by MS as compared to the HPTLC method, which is significant at 6 h and 24 h measurements (**Fig. 4C**). This could be explained either due to limited sample degradation in HPTLC samples or a matter of working on the edge of detection sensitivity. Additionally, we observed that scattering of individual data points is higher at earlier time points. This seems reasonable as during exponential growth small differences in growth can lead to substantial difference in lipid content, whereas arrival in stationary phase rather stabilises and compensates for potential differences.

In summary, our analysis suggests acceptable overall comparability between both methods for lipid analysis. However, with very stringent statistical testing of data pairs statistical difference can be detected for some lipid classes at critical time points.

### 5. & 6. Validation of HPTLC-based lipidomic method by quantification of example yeast strains with described lipidomic changes

The next step to fully validate the HPTLC-based lipidomics method was to measure and quantify total lipids of a set of yeast strains which have already been described to have significantly altered lipid profiles. We chose the *dga1*Δ *lro1*Δ *are1*Δ *are2*Δ (QKO) and the *pah1*Δ strain. The QKO has been used in several studies and completely lacks LDs, since neutral fat synthesis is completely abrogated when all four genes encoding acyltransferases are deleted [26, 36, 62]. *PAH1* on the other hand encodes the phosphatidate phosphatase called lipin [63]. The *pah1*Δ strain thus shows numerous changes in the lipid profile which we use as a reference to validate our data.

**Figure 5:**
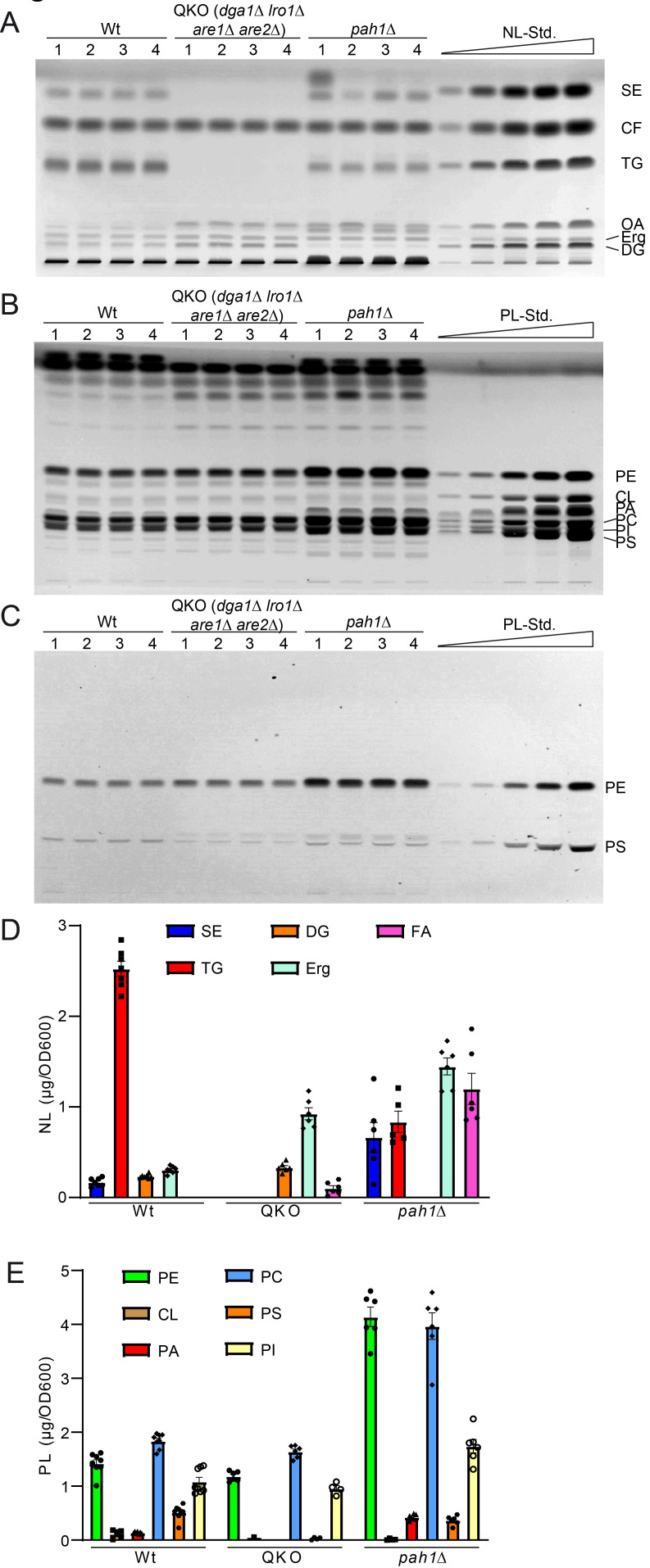
FIGURE 5: Further validation of HPTLC-based lipidomic method by quantification of yeast strains with known lipidomic changes. **(A)** HPTLC plate showing neutral lipid separation of wildtype (wt), *dga1*Δ *lro1*Δ *are1*Δ *are2*Δ (QKO), and *pah1*Δ each in quadruple. **(B, C)** HPTLC plate showing phospholipid separation, derivatised using Primulin **(B)** visualising all lipids and ninhydrin **(C)**, which visualizes PS and PE only. **(D, E)** Lipidomic quantification of neutral lipids **(D)** and phospholipid classes **(E)**.

**Figure 6:**
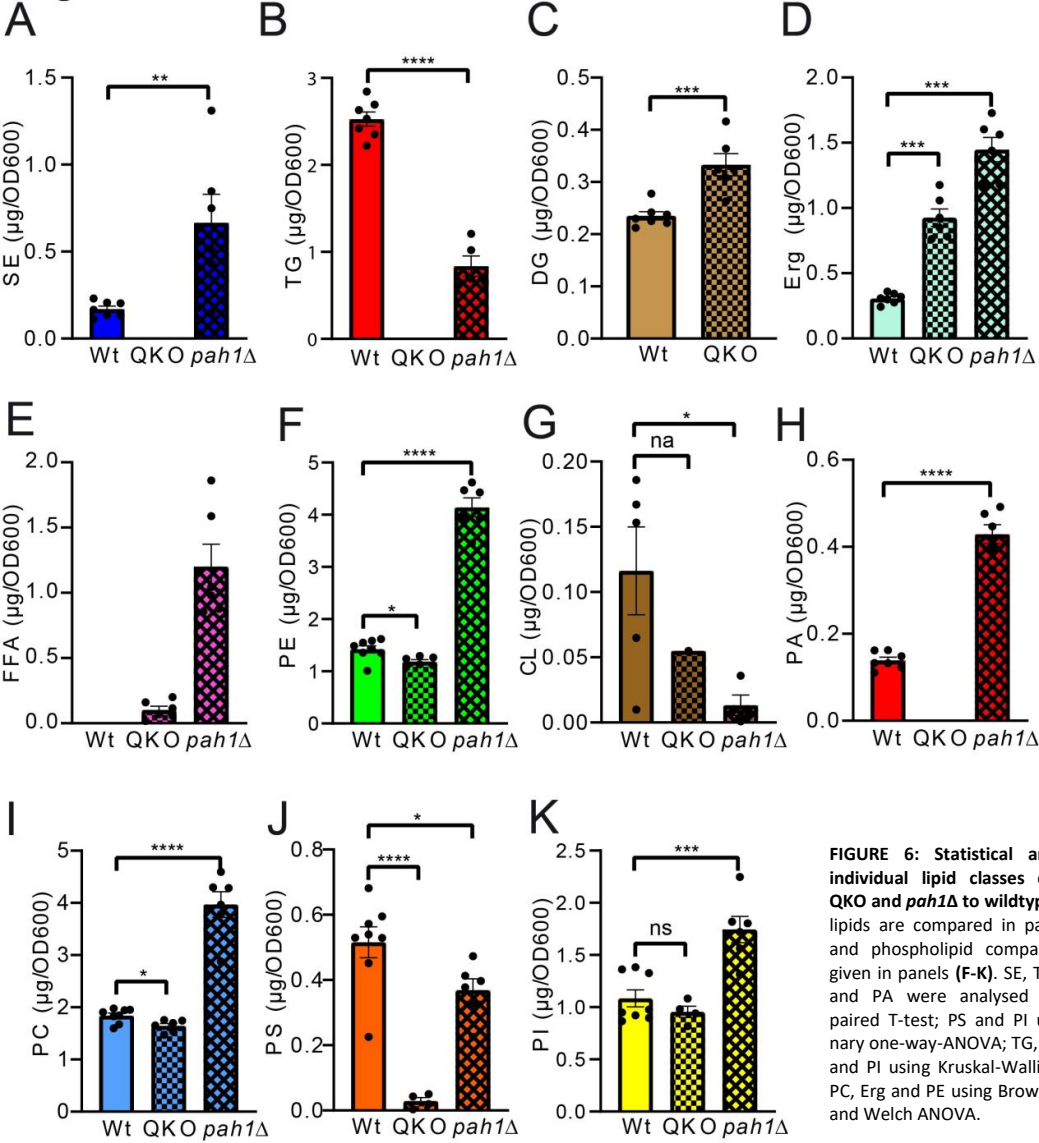
FIGURE 6: Statistical analysis of individual lipid classes comparing QKO and *pah1*Δ to wildtype. Neutral lipids are compared in panels **(A-E)** and phospholipid comparisons are given in panels **(F-K)**. SE, TG, DG, CL, and PA were analysed using unpaired T-test; PS and PI using ordinary one-way-ANOVA; TG, SE, PC, PS, and PI using Kruskal-Wallis test and PC, Erg and PE using Brown-Forsythe and Welch ANOVA.

Analysis of the QKO lipidome with the HPTLC method revealed the expected results, which are in line with previous descriptions of the knock out mutant (**Fig. 5** and **6**). We could confirm complete lack of the neutral lipids TG (**Fig. 6A**) and SE (**Fig. 6B**). For DG an increase was only observed as a non-significant trend (**Fig. 6C**), whereas free Erg was significantly increased (**Fig. 6D**). Since FA levels of the wildtype are at the detection limit (**Fig. 2A, 5A**, note 4), we only quantified FA for the QKO and *pah1*Δ mutants (**Fig. 5D, 6E**). Similarly, DG-levels of *pah1*Δ were beyond detection limit and excluded from statistical analysis (**Fig. 5D, 6C**). Regarding the phospholipids in the QKO we detected a slight but significant reduction of PE (**Fig. 6F**) and PC (**Fig. 6I**) whereas the decrease in PS was very strong and significant (**Fig. 6J**). PA was below detection level in the QKO (**Fig. 6H**), and a non-significant trend was observed for CL reduction (**Fig. 6G**).

The *pah1*Δ strain has been reported to have reduced levels of DG, TG, and PS whereas PA, PI and SE should be increased [63]. We could confirm all of these changes by our HPTLC-measurements (**Fig. 5 and 6**). Additionally, we observed significant increase of Erg, PE and PC (**Fig. 6**), which showed varying quantities depending on the time of analysis (exponential vs. stationary phase) in the formerly published analyses [63].

In summary we observed that all major changes in the lipid profile of QKO and *pah1*Δ in *S. cerevisiae* can be detected using HPTLC according to our here-described protocols. This suggests that the method is adequate for scientific lipid analysis in yeast especially when sample quantity is not a limiting factor.

## MATERIAL AND METHODS

### Laboratory Equipment

Pyrex glass tubes (30x100mm & 18x100mm)

Acid-washed glass beads (0.4 - 0.6 mm, VWR, LENZ05124005)

HPTLC plates (Sigma/Merck 1.05641.0001)

### Automatic equipment (for optimised results and reproducibility)

Heidolph Multi Reax shaker

Automatic TLC sample applicator (ATS 4, CAMAG, 022.7400)

Automatic developing chamber (ADC 2, CAMAG, 022.8380)

Derivatizer (CAMAG, 022.6000)

Filter paper for chamber saturation (CAMAG, 022.8371)

TLC plate heater (CAMAG, 022.3306)

CAMAG TLC Visualizer 2 (022.9810)

CAMAG TLC software visionCATS basic (028.0000)

CAMAG visionCATS Visualizer Enhanced Evaluation Package (028.2020)

### Manual equipment (low-cost alternative)

Filter paper (Whatman)

Twin trough glass thin-layer chromatography (TLC) developing chambers (CAMAG)

### Reagents

Acetic acid (Roth 3738.4), ROTIPURAN^®^ 100 %, p.a.

Acetone (Roth 9372.2), ROTIPURAN^®^ ≥99,8 %, p.a., ACS, ISO

Chloroform (Roth 4432.1), ROTISOLV^®^ ≥99,8 %, UV/IR Grade, stabilised

Diethylether (Roth 3942.6), ROTIPURAN^®^ ≥99,5 %, p.a., stabilised

Ethanol (Roth P075.4), 96 %, Ph. Eur., extra pure

Isopropanol (Sigma Aldrich 33539M), puriss. p.a., ACS reagent, reag. ISO, reag. Ph. Eur., ≥99.8% (GC)

Methanol (Roth 4627.2), ROTIPURAN^®^ ≥99,9 %, p.a., ACS, ISO

n-hexane (Roth 7573.1), ROTISOLV^®^ Pestilyse^®^ plus ≥99 %

n-heptane (Roth 7566.1), ROTISOLV^®^ Pestilyse^®^ plus ≥99 %

Petroleum ether (Roth T170.1), ROTISOLV^®^ Pestilyse^®^

Phosphatidylcholine 34:1 (Avanti Polar Lipids 850457P)

Phosphatidylethanolamine 34:1 (Avanti Polar Lipids 850757P)

Phosphatidylserine 34:1 (Avanti Polar Lipids 840034P)

L-α-phosphatidylinositol (Avanti Polar Lipids 840042P)

Diacylglycerol (16:0-18:1; Avanti Polar Lipids 800815O)

L-α-phosphatidic acid from chicken egg, mixture of fatty acids (16:0 (34.2 %), 16:1 (1 %), 18:0 (11.5 %), 18:1 (31.5 %), 18:2 (18.5 %), 20:4 (2.7 %), 22:6 (0.7 %); (Avanti Polar Lipids 840101P)

Cholesteryloleate (Avanti Polar Lipids 700269P)

Cardiolipin (18 : 1) (Avanti Polar Lipids 710335P)

Triolein (Avanti Polar Lipids 870110O)

Ergosterol (Acros Organics 117810050, see note 1)

Oleic acid (Sigma O1008)

Cholesterylformate (Sigma, S448532)

Primuline (Sigma 206865)

Ninhydrine spray reagent (Sigma N1286)

### Yeast strains and growth conditions

All experiments were carried out in BY4741 (*Mat***a** *his3*Δ1; *leu2*Δ0; *met15*Δ0*; ura3*Δ0) obtained from Euroscarf. The quadruple knock out mutant (QKO, BY4741), which was generated and described previously [26, 62], was genetically manipulated as follows: *ycr048w*Δ*::KanMX4 ynr019w*Δ*::KanMX4 yor245c*Δ*::KanMX4 ynr008w*Δ*::KanMX4*. The *pah1*Δ strain (also in the genetic background of BY4741) was a kind gift from Heimo Wolinski.

All experiments were carried out in synthetic complete medium with 2 % D-(+)glucose (Roth) with additional myo-inositol (SCD+Ino). SCD+Ino medium contains 0.17% yeast nitrogen base (BD Difco, 233520), 0.5% (NH_4_)_2_SO_4_ (Roth, 9218.1), 2% D-(+)glucose (Roth), 8 mg/L myo-inositol and amino acids, adenine and uracil according to **Table 1**.

**Table 1.** Amino acids, adenine and uracil.

**No.**	**Component**	**Final conc. (mg/l)**	**Distributor**	**Product no.**
1	L-alanine	30	SERVA	11482.02
2	L-arginine base	30	SERVA	13909.02
3	L-asparagine-monohydrate	30	SERVA	14110.02
4	L-aspartic acid	30	SERVA	14180.02
5	L-cystine	30	SERVA	17880.02
6	L-glutamine	30	SERVA	22942.02
7	L-glutamic acid	30	SERVA	23000.01
8	Glycine	30	SERVA	23390.02
9	histidine-Hcl monohydrate	80	SERVA	24842.02
10	L-isoleucine	30	SERVA	26540.03
11	L-leucine	200	SERVA	27690.02
12	L-lysine hydrochloride	30	Roth	1700.2
13	L-methionine	30	SERVA	28821.02
14	L-phenylalanine	30	SERVA	32191.02
15	L-proline	30	SERVA	33582.03
16	L-serine	30	SERVA	34962.03
17	L-threonine	30	SERVA	36382.03
18	L-tryptophan	30	SERVA	37422.03
19	L-tyrosine	30	SERVA	37540.03
20	L-valine	30	SERVA	38064.02
21	Uracil	320	Roth	7288.3
22	Adenine	30	SERVA	10739.02

All media were prepared with ultrapure water (MilliQ) and subsequently autoclaved (20 min, 121°C, 110 kPa). Amino acid mixture (including uracil and adenine) and glucose were sterilised separately as 10× stocks and added after autoclaving. Myo-inositol (Sigma, I5125) was added from a sterile filtered 10,000 x stock (80 g/L) after autoclaving. All yeast cultures were inoculated from a stationary overnight culture to an OD_600_ = 0.1 and then grown at 30°C and 145 rpm shaking for 6, 12 or 24 hours.

### Total yeast lipid extraction

In total, 80 OD_600_ units were harvested at indicated time points after inoculation. Total lipids were extracted with chloroform/methanol (CHCl_3_/MeOH) 2:1 (v/v) according to Folch *et al.* [64] and essentially as described before [65]. Samples were transferred into thick-walled glass tubes with screw caps (Pyrex 30 x 100 mm) and combined with 1 ml acid washed glass beads, 5 ml CHCl_3_/MeOH (2:1; v/v*)* and 125 µg cholesterylformate (CF) (Sigma, S448532) as internal standard. Subsequently cells were lysed by shaking in a Heidolph Multi Reax shaker at an intensity of 8 for 30 minutes. 1 ml of dH_2_O was added to each sample and samples were shaken for 10 more minutes. Subsequently, samples were centrifuged at 2500 rpm for 5 minutes and the aqueous phase was discarded. 2 ml of artificial upper phase consisting of methanol/H_2_O/chloroform (48/47/3; v/v/v) was added to the samples and samples were vortex-mixed. The washed samples were again centrifuged at 2500 rpm for 5 minutes to achieve phase separation. The aqueous upper phase was discarded and the organic phase at the bottom was collected using a Pasteur pipette. The entire organic phase was harvested avoiding any contamination of watery phase. The lipid extracts were transferred to fresh pyrex tubes (18 x 100 mm) and the solvent was evaporated completely under a stream of nitrogen. The dried lipid samples were dissolved in 1 ml chloroform/methanol (2:1; v/v), transferred to 1.5 ml glass vials with caps and stored at -20°C.

### Quantification by high-performance thin-layer chromatography

For neutral lipid and phospholipid separation a total of 20 µL of lipid extracts was applied on HPTLC silica gel 60 plates, 20 x 10 cm (Merck, 1.05641.001) using a CAMAG automatic TLC sampler (ATS4). Lipid separation was performed using a CAMAG automatic developing chamber (ADC2). Neutral lipids were separated with n-hexane, n-heptane, diethylether, acetic acid (63/18.5/18.5/1; v/v/v/v) as mobile phase [58], whereas phospholipid separation was carried out using CHCl_3_/MeOH/water (32.5:12.5:2; v/v/v) mixture as mobile phase [49, 53, 54]. HPTLC plates were derivatized with 0.01% primuline (dissolved in 80% acetone) applied in a CAMAG derivatizer followed by mild heating to 40°C for 2 minutes on a CAMAG TLC plate heater 3. Developed HPTLC plates were imaged using a CAMAG TLC visualizer 2 with VisionCATS software. Since peak separation of PI and PS was not ideal in all samples, we conducted an additional derivatisation step with ninhydrin spray reagent (Sigma Aldrich, N1286), which only stains phospholipids containing free amino groups and thus allows quantification of PS without PI. HPTLC bands were processed into chromatograms and quantified by polynomial regression of standard curves calculated from the applied standards. For phospholipids the standard contained l-α-phosphatidylinositol (840044P), phosphatidylcholine (16:0-18:1; 850457P), phosphatidylethanolamine (16:0-18:1; 850757P), phosphatidylserine (18:1-18:1; 840034P), cardiolipin (18:1-18:1; 710335P), phosphatidic acid (16:0 (34.2%), 16:1 (1%), 18:0 (11.5%), 18:1 (31.5%), 18:2 (18.5%), 20:4 (2.7%), 22:6 (0.7%); 840101P) each at 500 ng/µl all purchased individually from Sigma Aldrich. The PL-standard was dissolved in chloroform/methanol (2/1). As a neutral lipid standard, we used a custom-made neutral lipid standard consisting of a mix of cholesteryl-oleate (700269P), cholesterylformate (S448532), triolein (870110O), diacylglycerol (16:0-18:1); 800815O)), oleic acid (O1008) all purchased individually from Sigma Aldrich, and ergosterol from Thermofisher Scientific (117810050) each at 500 ng/µl. The NL-standard was dissolved in heptane/isoproanol (1/1). Both standards were applied at increasing quantities for phospholipids from 0.25 µg to 10 µg; for neutral lipids 0.5-10 µg absolute mass.

### Lipid quantification by shotgun lipidomics

Shotgun lipidomics were performed by the company Lipotype on a commercial basis following standard protocols [2]. Basic analysis of yeast cells, covering nine of the most important lipid classes was performed, which included PA, PC, PE, PG, PI, PS, DG, TG, and EE. Additionally, CL quantification was selected. The original report is accessible at Mendeley data (doi: 10.17632/gf9z9ky3h5.1).

### Statistical analysis

Tests for statistical significance were performed using GraphPad Prism 8.4.3. The obtained data from HPTLC and MS analyses were processed as paired data, since they were generated from exactly the same samples. Normal distribution of datasets was assessed using Shapiro-Wilk tests. In case of negative Shapiro-Wilk test a ROUT analysis with Q=10% to identify likely outliers was conducted and cleaned datasets were used for further statistical analysis. Repeated measure (RM) one-way ANOVA with Sidak's multiple comparisons test was conducted if no missing values were present; for datasets containing missing values a mixed effects analysis with Holm-Sidak's multiple comparisons test was performed. All original GraphPad Prism files are accessible at Mendelay data.

Pearson correlation analysis of lipid species by HPTLC and MS measurements was conducted using R (version 4.3.2.; **Table 2**). Data was imported from MS Excel via the readxl package [66], cleaned and transformed using the packages dplyr [67], stringr [68] and tidyr [69] from the tidyverse collection. Correlation calculations were performed with the rstatix package [70]. Packages ggplot2 [71], RColorBrewer [72] and pheatmap [73] were used for plotting.

**Table 2.** R packages used in this study.

**Package**	**Version**
dplyr [67]	1.1.4
ggplot2 [71]	3.3.4
pheatmap [73]	1.0.12
RColorBrewer [72]	1.1-3
readxl [66]	1.4.3
rstatix [70]	0.7.2
stringr [68]	1.5.1
tidyr [69]	1.3.0

### Notes

1) The analysis of Erg could possibly be improved using an alternative source as ergosterol standard. It seems like there is substantial impurity in the Erg which was used here, which has an impact on the precise estimation of Erg levels.

2) Inositol is not sufficient in standard SC medium [74], which is why it was additionally provided as a supplement.

3) Erg quantification was not included in the MS-approach by lipotype, which is why a comparison for Erg is not applicable. MS-based quantification of Erg and sterolesters is generally difficult because stable-isotope-labelled compounds are not available.

4) The analysis of FA in wildtype which is at detection limit could be overcome by increasing total lipid amounts for application. This can be achieved easily by evaporation of total lipid extracts under a stream of nitrogen and resolubilisation in less solvent.

### Data availability

The original data are accessible at Mendeley Data: Meyer, Thorsten; Knittelfelder, Oskar; Rockenfeller, Patrick (2023), “Quantifying yeast lipidomics by high-performance thin-layer chromatography (HPTLC) and comparison to mass spectrometry-based shotgun lipidomics.”, Mendeley Data, V1, doi: 10.17632/gf9z9ky3h5.1

## AUTHOR CONTRIBUTION

Conceptualisation PR; Methodology, TM, OK and PR; Validation, TM, and PR; Formal Analysis, TM, MS and PR; Investigation, TM; Data Curation, MS and PR; Writing-Original Draft Preparation, PR; Writing-Review and Editing, TM, OK, and MS; Figure Visualisation, PR and MS; Supervision, Project Administration, and Funding Acquisition, PR. All authors have read and agreed to the published version of the manuscript.

## SUPPLEMENTAL MATERIAL

Click here for supplemental data file.

All supplemental data for this article are available online at www.microbialcell.com/researcharticles/2024a-meyer-microbial-cell/.

## Abbreviations:

CF: – cholesterylformate;
CL: – cardiolipin;
DG: – diglyceride;
Erg: – ergosterol;
FA: – fatty acid;
LD: – lipid droplet;
HPTLC: – high-performance TLC;
MS: – mass spectrometry;
OA: – oleic acid;
PA: – phosphatidic acid;
PC: – phosphytidylcholine;
PE: – phosphatidylethanolamine;
PI: – phosphatidylinositol;
PL: – phospholipid;
PS: – phosphatidylserine;
QKO: – quadruple knock out;
SE: – sterol ester;
TG: – triglyceride;
TLC: – thin-layer chromatography.
