# Supplementary material for: Quantifying yeast lipidomics by high-performance thin-layer chromatography (HPTLC) and comparison to mass spectrometry-based shotgun lipidomics
Source: Microb Cell. 2024 Feb 21;11:57–68. doi: 10.15698/mic2024.02.815 (PMC10879857; doi:10.15698/mic2024.02.815)
Supplement: Supplementary file 1 [file mic-11-057-s01.pdf]

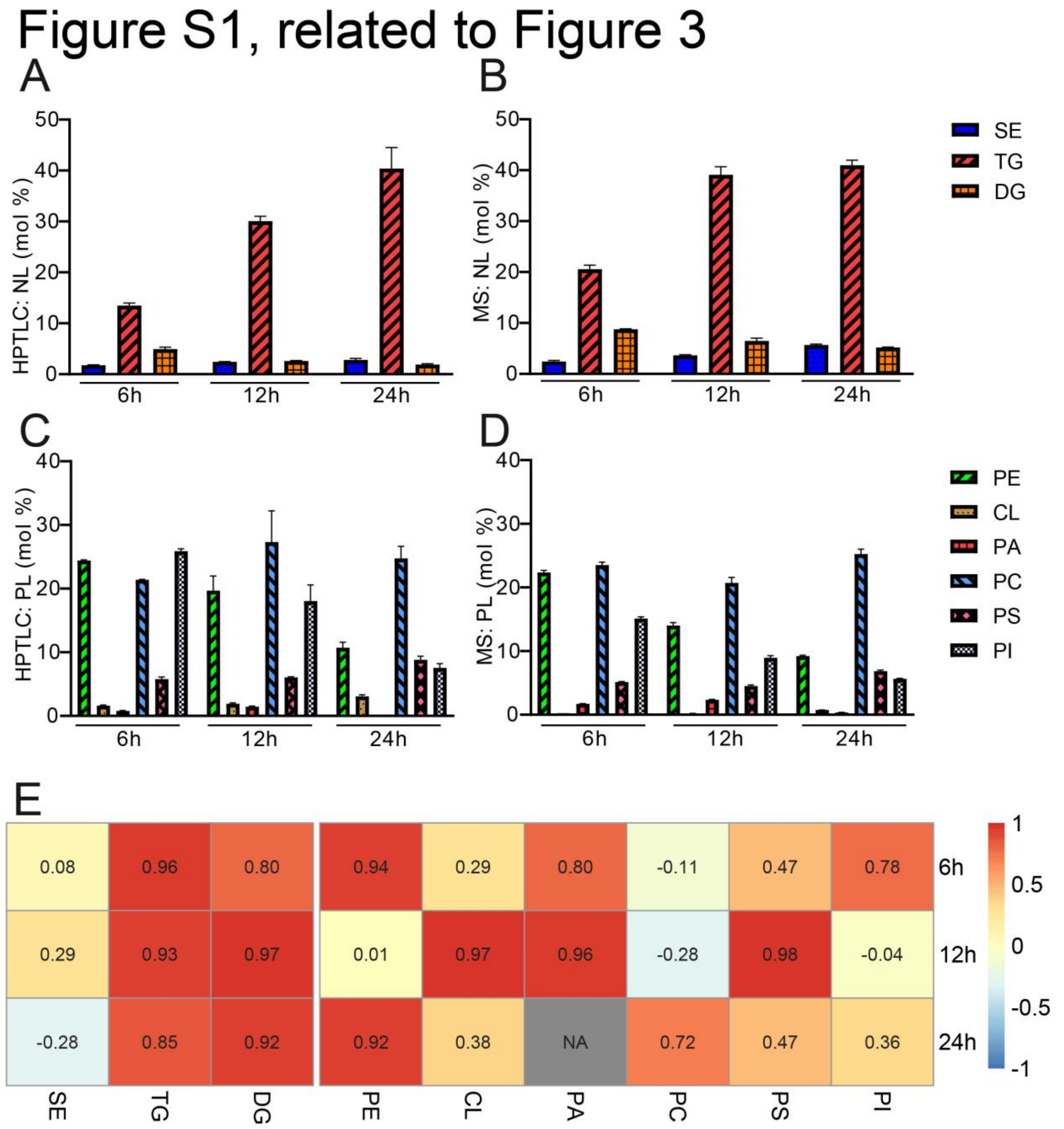

Figure S1: Relative quantification of HPTLC-lipidomics and MS-assisted shotgun lipidomics.

(A, B) HPTLC-derived neutral lipidomic quantification **(A)** and analysis of the same samples by MS-based shotgun lipidomics **(B)** deliver a comparable overall picture of neutral lipid content in yeast. **(C, D)** HPTLC-derived phospholipidomic quantification **(C)** and analysis of the same samples by shotgun lipidomics **(D)** deliver a comparable overall picture of the major phospholipid classes in yeast. **(E)** Pearson correlation analysis comparing HPTLC with MS results. Related to Figure 3.

Figure S2, related to Figure 3

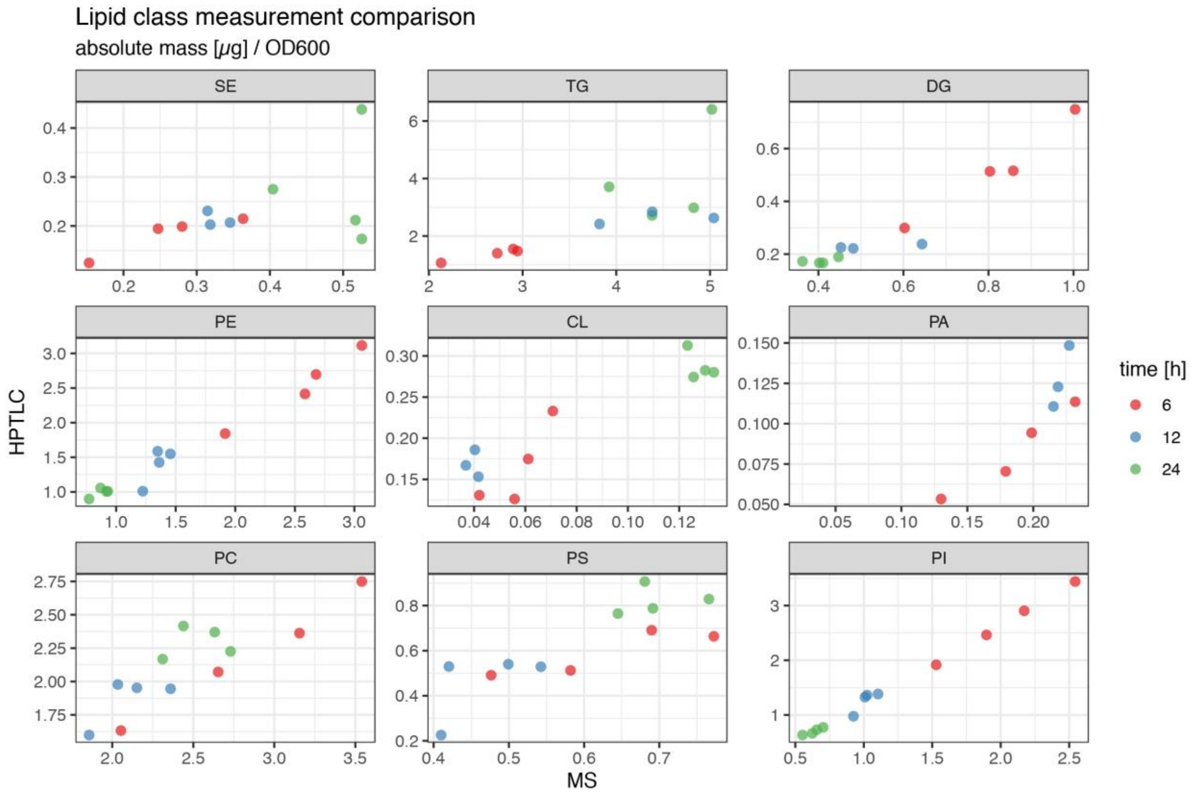

Figure S2: Comparison of lipid class measurements.

Individual data pairs are shown in a scatter plot to support the Pearson correlation analysis. Related to Figure 3.

Figure S3, related to Figure 4

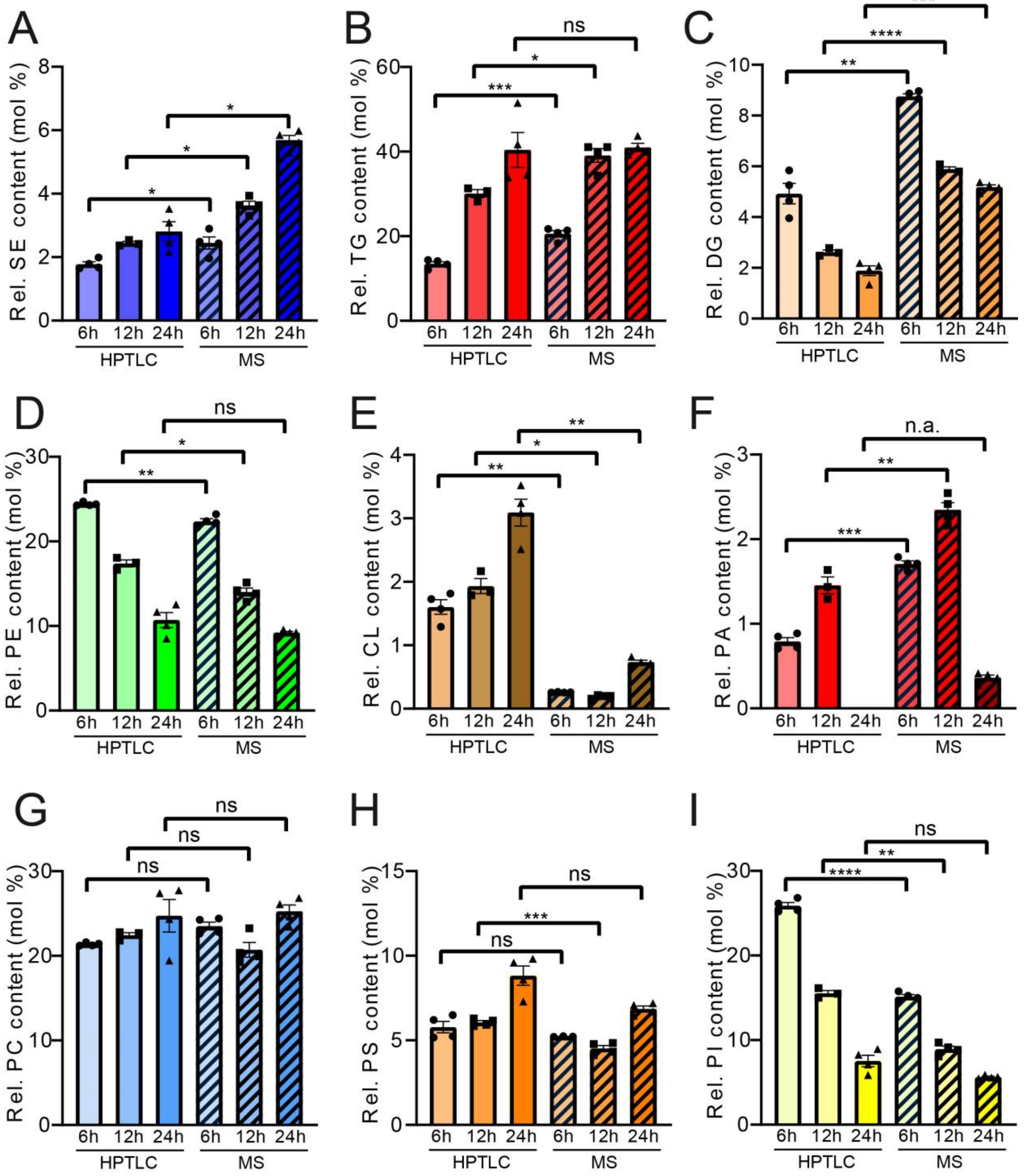

Figure S3: Paired statistical analysis of individual lipid classes (relative) for HPTLC-lipidomics vs. MS-assisted shotgun lipidomics reveals differences between both methods. Neutral lipids are compared in panels **(A-C)** and phospholipid comparisons are given in panels **(D-I)**. Statistical analysis was performed using paired tests: TG, DG, SE, CL and PA were analysed using mixed effects analysis with Sidaks´s multiple comparisons test; PE, PC, PS, and PI were analysed using RM ANOVA with Sidak´s multiple comparisons test. Related to Figure 4.
